# Treatment With Bilevel PAP Is Associated With a Reduction in Severe Exacerbations in COPD-OSA Overlap: A Retrospective Claims Analysis

**DOI:** 10.1016/j.chpulm.2024.100114

**Published:** 2024-10-28

**Authors:** Daniela Téllez, Ann Cameron, Fatima Sert-Kuniyoshi, Peter Cistulli, Jean Louis Pépin, Adam V. Benjafield, Atul Malhotra, medXcloud Group, Victoria M. Pak

**Affiliations:** a ResMed Science Center, San Diego, CA; b Charles Perkins Centre, Faculty of Medicine and Health, University of Sydney, Sydney, NSW, Australia; c Department of Respiratory and Sleep Medicine, Royal North Shore Hospital, Sydney, NSW, Australia; d University Grenoble Alpes, INSERM, CHU Grenoble Alpes, HP2 Laboratory, Grenoble, France; e ResMed Science Centre, Sydney, NSW, Australia; f Department of Medicine, University of California at San Diego, La Jolla, CA; g Nell Hodgson Woodruff School of Nursing, Rollins School of Public Health, Emory University, Atlanta, GA

**Keywords:** COPD, OSA, overlap syndrome, disease exacerbation, intermittent positive airway pressure, noninvasive ventilation

## Abstract

**Background:**

There are no guidelines for OSA assessment in patients with COPD. Home noninvasive ventilation (NIV) studies have excluded patients with comorbid OSA. Thus, it is unclear whether home NIV is associated with reduced exacerbation risk in patients with overlap syndrome.

**Research Question:**

Does home NIV impact the rate of severe exacerbations in patients with overlap syndrome 1 year after therapy initiation?

**Study Design and Methods:**

A retrospective analysis was performed on administrative claims data from patients with COPD and OSA who received an NIV device claim between 2015 and 2020. Patients were characterized 1 year before NIV initiation and 1 year after NIV initiation. A modified Poisson regression model was built to identify predictors for severe exacerbation occurrence during follow-up.

**Results:**

A total of 23,992 patients were included in the analysis (mean age, 61.3 ± 10.1 years; 44.9% female). The proportion of patients with ≥ 1 severe exacerbation was 10.2% in the year before NIV initiation and 5.9% in the year after NIV initiation (χ^2^ = 440.5; *P* < .0001). Occurrence of a severe exacerbation in the year prior to NIV was associated with a nearly five-fold higher risk of severe exacerbation during follow-up (risk ratio, 4.91; 95% CI, 4.39-5.48; *P* < .0001). Heart failure, pneumonia, and anxiety were the comorbidities most associated with increased severe exacerbation risk.

**Interpretation:**

To our knowledge, this is the first study to describe risk factors for severe exacerbations and to examine home NIV claims in this specific population. Results may be informative for overlap syndrome management, especially for preventing a first severe exacerbation and for the treatment of OSA as part of COPD management. Additional information is needed to optimize the access, timing, and benefits of NIV treatment in patients with overlap syndrome.

**Take-Home Points**

**Study Question:** Does home noninvasive ventilation (NIV) impact the rate of severe exacerbations in patients with overlap syndrome 1 year after therapy initiation?

**Results:** Using a large population sample of claims data from patients with COPD-OSA overlap syndrome, analyses showed that severe COPD exacerbations were significantly lower 1 year after NIV initiation compared with 1 year before NIV among patients with overlap syndrome (χ^2^ = 440.5; *P* < .0001). Risk factors for severe COPD exacerbation occurrence among patients with overlap syndrome do not differ significantly from those among COPD alone, with previous exacerbations being the greatest risk factor for future exacerbations (risk ratio, 4.91; 95% CI, 4.39-5.48; *P* < .0001). Heart failure, pneumonia, and anxiety were the comorbidities most associated with increased severe exacerbation risk.

**Interpretation:** With little data available on the impact of home NIV in patients with overlap syndrome, this study offers real-world insights into the benefits of NIV (bilevel positive airway pressure therapy) in this population. Results may be informative for overlap syndrome management, especially for preventing a first severe exacerbation and for the treatment of OSA as part of COPD management. Additional information is needed to optimize the access, timing, and benefits of NIV treatment in patients with overlap syndrome.

The simultaneous presence of COPD and OSA, known as overlap syndrome, has been described as a disease “greater than the sum of its parts”^1^ due to the amplified inflammatory and oxidative stress pathways generated by nocturnal hypoxemia from apneic events during sleep. The overlap in pathophysiology of each condition creates a worsening bidirectional prognostic relationship^2^ that is associated with increased risk of COPD-related exacerbations, systemic hypertension, cardiovascular disease, and mortality compared with COPD alone.^3^, ^4^, ^5^, ^6^ Prevalence estimates for overlap syndrome are limited in part due to differing diagnostic criteria,^4^ but COPD and OSA prevalence rates are on the rise worldwide,^7^ and as many as two-thirds of patients with COPD have been reported to have comorbid OSA.^3^

Recommended treatment for people with overlap syndrome is often determined by the predominant phenotype they exhibit.^4^ For example, patients with obesity-hypoventilation or sleep-related hypoventilation in COPD may be provided with bilevel positive airway pressure (PAP) therapy or high-intensity noninvasive ventilation (NIV), respectively.^4^ CPAP has also been shown to reduce lung hyperinflation^8^ and both COPD-related^9^^,^^10^ and all-cause hospitalizations^11^ in patients with overlap syndrome. Through additional pressure support, home NIV in the form of bilevel PAP may offer improved outcomes by treating OSA and alleviating COPD symptoms (eg, work of breathing, daytime gas exchange).^12^ However, there are no formal guidelines specifying approaches to test for or treat OSA in patients with COPD,^3^ and most data in this patient population are focused on CPAP only. Many randomized controlled trials (RCTs) of home-based NIV treatment in patients with COPD have excluded patients with OSA or did not assess for comorbid sleep apnea.^13^ Therefore, it remains unclear whether home NIV (bilevel PAP) is associated with reduced exacerbation risk in patients with overlap syndrome.

Bilevel PAP is delivered through spontaneous or spontaneous-timed modes through flow-generator devices coded E0470 and E0471 under the Healthcare Common Procedure Coding System (HCPCS). The American Thoracic Society and European Respiratory Society clinical guidelines^14^, ^15^, ^16^ recommend nocturnal NIV for individuals who have COPD with hypercapnic chronic respiratory failure. However, there is still uncertainty about the clinical benefits of home-based NIV treatment for patients with COPD in the United States due to mixed results from RCTs and lack of data reflecting US clinical practice and implementation. Vasquez et al^17^ showed that most patients with COPD are not on any form of home-based NIV therapy, with only 7.5% receiving home NIV (CPAP, bilevel PAP, or home NIV); of those, only 1.5% were receiving bilevel PAP. Fortis et al^18^ further highlight the underutilization of home NIV among patients with COPD and chronic hypercapnic respiratory failure after a COPD-related hospitalization.

Severe exacerbations are the greatest driver of COPD-related expenditures.^19^ Therefore, preventing exacerbations is the top priority in COPD research. However, risk factors for exacerbation have not been clearly determined,^20^, ^21^, ^22^ in part due to the multimorbidity and systemic heterogeneity of the disease.^23^ Individuals with overlap syndrome and untreated sleep apnea are more likely to experience severe COPD-related exacerbations.^24^

This analysis investigated the occurrence of severe COPD exacerbations (ie, exacerbations requiring hospitalization) before and after home NIV (bilevel PAP) setup, and aimed to identify risk factors associated with severe exacerbations in the year after NIV initiation in patients with COPD-OSA overlap syndrome.

## Study Design and Methods

### Data Source

This retrospective observational study used deidentified payer-sourced adjudicated claims data from a single cohort of patients with dispersed health plans in the United States (Inovalon Insights, LLC). Claims are presented through the International Classification of Diseases, 10th Revision (ICD-10), Current Procedural Terminology, and HCPCS codes for diagnoses, procedures, and devices billed to the patient.^25^^,^^26^ The study design was reviewed by an institutional review board (Advarra, reference No. Pro0004005) and deemed exempt from oversight.

### Study Population

**Figure 1:**
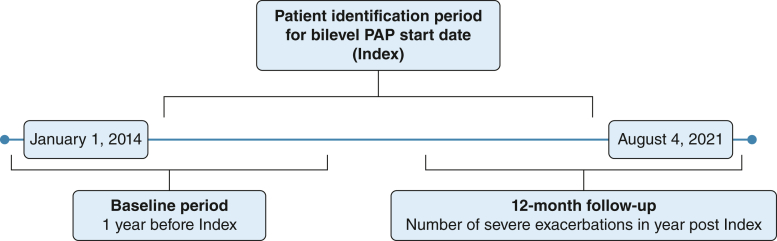
Patient identification period. PAP = positive airway pressure.

Patients who had a prescription for a home NIV device (HCPCS code E0470 or E0471) were included. The earliest service date between January 1, 2015, and August 4, 2020 (Fig 1), with a NIV claim was defined as the index date. This study population was restricted to patients who had a COPD diagnosis (ICD-10 codes J41-J44) and an OSA diagnosis (ICD-10 code G47.33) in the year before NIV device setup. Additional coding for chronic respiratory failure or blood gas conditions was not available for this analysis. Patients with an NIV claim who did not have a COPD or OSA diagnosis in the year before the index date were excluded. Available demographic data (age, biological sex, region, payer), diagnosis claims, comorbid conditions, medications, and health care utilization data were included for all patients 1 year before the index date and 1 year after the index date (ie, follow-up period). Any null or unknown data fields were excluded for demographic data.

### Outcomes

Severe exacerbations were defined as hospitalizations related to respiratory symptoms, as used in Mapel et al.^27^ Severe exacerbation occurrence was defined as a dichotomous outcome variable where 0 related to no severe exacerbations and 1 related to ≥ 1 severe exacerbation during follow-up. Relative risks for the occurrence of severe exacerbation events during follow-up were estimated for significant predictors in the selected model.

### Covariates

Twenty-nine comorbid conditions were included as potential risk factors. Demographic variables included age, sex, US region, and insurance payer type at the index date. Moderate and severe exacerbation occurrence in the year before NIV setup were also included as covariates, defined as doctor or emergency department visit with diagnosis codes and a prescription of drugs commonly used for COPD exacerbations (moderate exacerbation) or respiratory-related hospitalization (severe exacerbation).^27^ The variables for all listed comorbidities were coded as present if there was a qualifying ICD-10 diagnosis code for the condition in the year before the index. Additional covariates included were obesity status, COPD-related prescription medications, and level of COPD complexity, defined as the occurrence of claims for respiratory-related comorbid conditions or medical procedures (e-Table 1).^28^

### Statistical Analysis

All 23,992 observations were used for model development and assessment. A modified Poisson regression approach was used to obtain estimates of the relative risks, first described by Zou.^29^ The estimation of risk ratios (RRs) is preferred over ORs because they are easier to interpret and apply into clinical practice and ORs tend to overestimate event occurrence in cohort studies.^30^ Zou’s use of Poisson regression used in this study has been shown to estimate relative risk consistently and efficiently in cohort studies by providing similar estimates obtained by the Mantel-Haenszel procedure.^31^ Univariable analysis was conducted to examine the association between each available covariate and the occurrence of severe exacerbation during follow-up. Predictors identified as statistically significant in the univariate analysis were included in the model selection process. A stepwise logistic regression analysis was then used to arrive at the final model. The selected model included only statistically significant predictors. Using these selected covariates, a multivariate analysis was conducted to estimate adjusted RR values through the modified Poisson regression approach. RR values are reported with 95% CIs and *P* values. Statistical analyses were performed using SAS version 9.4 (SAS Institute).

## Results

### Study Population

**Figure 2:**
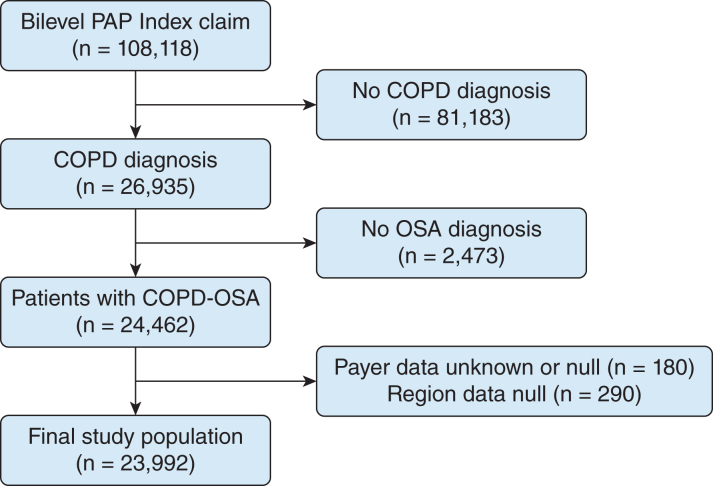
Consolidated Standards of Reporting Trials diagram. PAP = positive airway pressure.

A total of 108,118 patients had a qualifying NIV index date. Most patients with a prescribed NIV device did not have a COPD diagnosis (75.1%), and most patients with COPD had comorbid OSA (90.8%). The final study population included 23,992 patients with COPD-OSA overlap syndrome and an NIV claim (Fig 2). Forty-five percent of patients were female, and the mean age was 61.3 ± 10.0 years (Table 1). Over one-half of patients were categorized as morbidly obese and had moderate COPD complexity.

**Table 1:** Baseline Clinical and Demographic Characteristics (N = 23,992)

Characteristic	Value
**Male sex**	13,213 (55.07)
**Age, y**	61.3 [10.0]
40-44	879 (3.66)
45-54	5,148 (21.46)
55-64	10,279 (42.84)
65-69	2,702 (11.26)
≥ 70	4,984 (20.77)
**Payer at index**	
Medicaid	8,419 (35.09)
Medicare Advantage	7,843 (32.69)
Commercial	7,730 (32.22)
**Region**	
Midwest	7,663 (31.94)
Northeast	3,745 (15.61)
South	7,803 (32.52)
West	4,781 (19.93)
**COPD complexity**	
Low	7,187 (27.16)
Moderate	13,441 (50.79)
High	5,837 (22.06)
**Obesity status**	
Not categorized/healthy	4,384 (18.27)
Overweight	988 (4.12)
Obese	5,468 (22.79)
Morbidly obese	12,895 (53.75)
**Comorbidities**	
Anxiety	7,837 (32.67)
Asthma	8,992 (37. 48)
Atrial fibrillation	4,818 (20.08)
Atrial flutter	1,026 (4.28)
CSA	1,950 (8.13)
Cancer	2,897 (12.07)
Cerebrovascular disease	3,334 (13.90)
COVID-19	1,877 (7.82)
CAD	10,103 (42.11)
Dementia	638 (2.66)
Depression	9,040 (37.68)
Fatigue	6,462 (26.93)
GERD	10,640 (44.35)
Heart failure	9,929 (41.38)
Hyperlipidemia	17,671 (73.65)
Hypertension	21,252 (88.58)
Insomnia	4,365 (18.19)
Other arrhythmia	4,518 (18.83)
Other mood disorders	2,388 (9.95)
Pneumonia	5,675 (23.65)
Psychotic disorders	2,392 (9.97)
Secondary pulmonary hypertension	3,948 (16.46)
Snoring	4,177 (17.41)
Somnolence	1,356 (5.65)
Stroke	1,441 (6.01)
Type 1 diabetes	1,560 (6.50)
Type 2 diabetes	13,386 (55.79)
**Diagnosis associated with NIV claim**	
COPD	3,480 (14.50)
SDB	21,921 (91.37)
**CPAP claim present 1 y before NIV**	5,693 (23.73)
**NIV HCPCS code**	
E0470	20,877 (87.02)
E0471	3,115 (12.98)
**Medications**	
Maintenance^a^	12,644 (52.70)
Rescue^b^	14,991 (62.50)
**Annual health care costs, US $**^c^	
1 y before NIV initiation	25,249.13 [34,018.39]
1 y after NIV initiation	24,697.80 [32,325.38]

Data are presented as mean [SD] or No. (%). CAD = coronary artery disease; CSA = central sleep apnea; GERD = gastroesophageal reflux disease; HCPCS = Healthcare Common Procedure Coding System; ICS = inhaled corticosteroid; LABA = long-acting β-agonist; LAMA = long-acting muscarinic antagonist; NIV = noninvasive ventilation; SDB = sleep disordered breathing.

a Maintenance medications = monotherapy (LABA, LAMA, ICS, phosphodiesterase-4 inhibitor, xanthine derivative) + combination therapy (LABA + LAMA, LABA + ICS, LABA + LAMA + ICS, or other combination).

b Rescue medications = short-acting β-agonist, short-acting muscarinic antagonist, or short-acting β-agonist + short-acting muscarinic antagonist.

c Total estimated payment for all claims (medical and prescription) per year.

### Impact of Bilevel PAP

**Figure 3:**
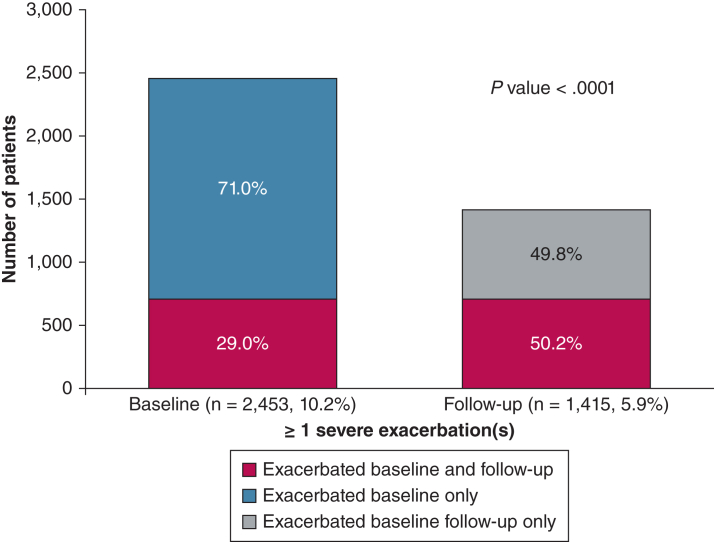
Severe exacerbations occurrence distribution. At baseline, 2,453 patients (10.22% of study population) had at least 1 severe exacerbation, whereas 1,415 (5.90% of study population) patients had at least 1 severe exacerbation during follow-up. Baseline = 1 y prior to noninvasive ventilation index; Follow-up = 1 y after noninvasive ventilation index.

**e-Figure 1:**
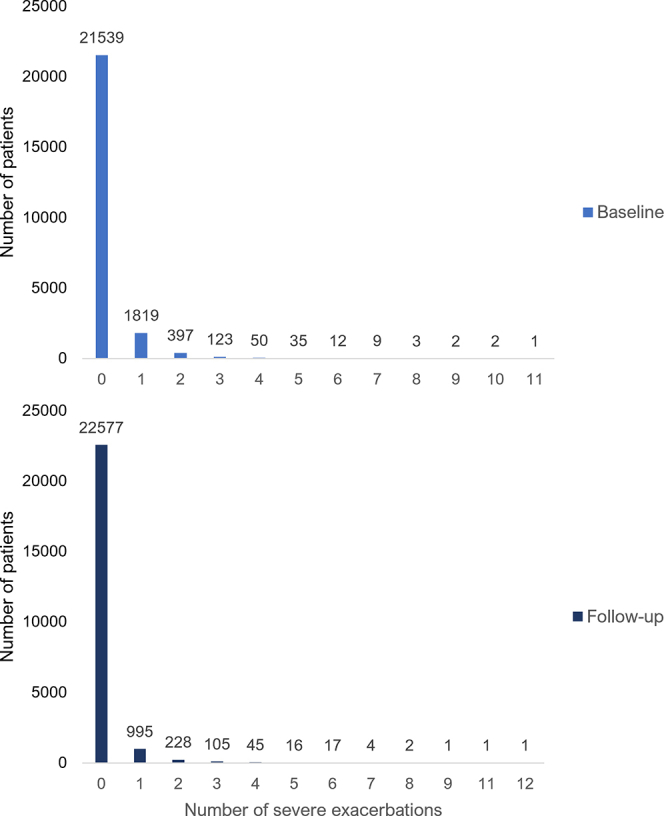


Most patients did not experience any severe exacerbations during the study period (e-Fig 1). On average, unadjusted health care utilization costs were higher before NIV initiation than during postindex follow-up. Over 10% of patients had ≥ 1 severe exacerbation in the year before NIV and 5.89% had ≥ 1 severe exacerbation during follow-up (McNemar’s χ^2^ = 440.5, *P* < .0001) (Fig 3). Of those who experienced severe exacerbations at follow-up, nearly one-half did not exacerbate in the year before NIV (e-Table 2).

### Univariate Risk Analysis

In the unadjusted analysis, the occurrence of a severe exacerbation before NIV setup was by far the strongest predictor of risk for a future exacerbation (RR, 8.87; 95% CI, 8.06-9.76). High COPD complexity, being a Medicaid beneficiary, and pneumonia in the year before NIV were associated with an increased risk of severe exacerbation occurrence during follow-up (RR, 6.29; 95% CI, 5.02-7.89; RR, 3.11; 95% CI, 2.69-3.59; and RR, 3.11; 95% CI, 2.81-3.43, respectively).

### Adjusted Risk Estimates

**Figure 4:**
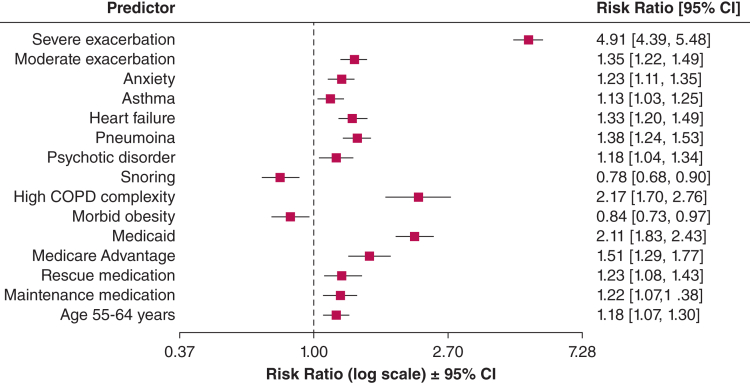
Adjusted risk ratio values for severe exacerbation occurrence.

All predictors in the selected model were statistically significant and indicated increased risk for severe exacerbation occurrence, except for snoring and morbid obesity that showed a protective effect against exacerbations using this model. Occurrence of a severe exacerbation in the year before NIV remained the strongest predictor of exacerbation during follow-up, with a nearly 5-fold increased risk for future severe exacerbation occurrence in the adjusted model (Table 2). Like the unadjusted model, both high COPD complexity and Medicaid coverage were associated with more than double the risk of severe exacerbation in the year after NIV setup (RR, 2.17; 95% CI, 1.70-2.76; RR 2.11; 95% CI, 1.83, 2.43, respectively). Occurrence of a moderate exacerbation was associated with 35% higher risk of a severe exacerbation (RR, 1.35; 95% CI, 1.22-1.49). Comorbidities associated with increased risk of severe exacerbation included pneumonia, heart failure, anxiety, psychotic disorder, and asthma, in that order (Table 2). COPD maintenance and rescue medications were associated with an approximately 23% higher risk of severe exacerbation (RR, 1.22; 95% CI, 1.07-1.38, and RR, 1.23; 95% CI, 1.06-1.43, respectively). In terms of demographic characteristics, the risk of a severe exacerbation was significantly higher in the 55 to 64 year vs 40 to 44 year age group (RR, 1.18; 95% CI, 1.07- 1.30), and in those 55 to 64 years of age vs older individuals (who showed lower risk in the unadjusted model). Conversely, snoring was associated with 22% reduction in severe exacerbation risk (RR, 0.78; 95% CI, 0.68-0.90), whereas morbid obesity and obesity claims both reduced the risk of severe exacerbation by about 16% (RR, 0.84; 95% CI, 0.73-0.97, and RR, 0.84; 95% CI, 0.71-0.99, respectively). Significant predictors of severe exacerbation in patients with COPD prescribed home NIV therapy are summarized in Figure 4.

**Table 2:** Risk for Severe Exacerbation Occurrence Associated With Selected Model Predictors

Baseline Predictor	Unadjusted	Adjusted
Severe exacerbation	8.87 (8.06-9.76); < .0001	4.91 (4.39-5.48); < .0001
Moderate exacerbation	2.25 (2.03-2.49); < .0001	1.35 (1.22-1.49); < .0001
Anxiety	1.87 (1.69-2.08); < .0001	1.23 (1.11-1.35); < .0001
Asthma	1.67 (1.51-1.85); < .0001	1.13 (1.03-1.25); .0127
Heart failure	2.16 (1.95-2.40); < .0001	1.33 (1.20-1.49); < .0001
Pneumonia	3.11 (2.81-3.43); < .0001	1.38 (1.24-1.53); < .0001
Psychotic disorder	1.86 (1.63-2.13); < .0001	1.18 (1.04-1.34); .0080
Snoring	0.70 (0.60-0.82); < .0001	0.78 (0.68-0.90); .0006
COPD complexity (Ref: low)		
High	6.29 (5.02-7.89); < .0001	2.17 (1.70-2.76); < .0001
Moderate	1.12 (1.00-1.25); .0480	0.97 (0.87-1.07); .5070
Obesity (Ref: not categorized)		
Overweight^a^	1.22 (0.92-1.62); .1738	1.08 (0.83-1.41); .5638
Obese	0.94 (0.79-1.12); .5056	0.84 (0.71-0.99); .0426
Morbidly obese	1.46 (1.27-1.69); < .0001	0.84 (0.73-0.97); .0157
Payer at index (Ref: commercial)		
Medicaid	3.11 (2.69-3.59); < .0001	2.11 (1.83-2.43); < .0001
Medicare Advantage	1.74 (1.49-2.04); < .0001	1.51 (1.29-1.77); < .0001
Medication Use		
Rescue	2.75 (2.40-3.14); < .0001	1.23 (1.06-1.43); .0062
Maintenance	2.33 (2.08-2.61); < .0001	1.22 (1.07-1.38); .0016
Age, y (Ref: 40-44)		
55-64	1.31 (1.18-1.45); < .0001	1.18 (1.07-1.30); .0012

Values are risk ratio (95% CI); *P* value. Ref = reference.

a Overweight is defined as ICD code E66.3 and body-mass index (BMI) codes in the range 25 to 29.9.

## Discussion

This observational analysis provides a real-world understanding of risk factors associated with severe exacerbations in patients with COPD-OSA overlap syndrome using home NIV therapy. There was a significantly lower number of severe exacerbations in the year after NIV initiation compared with the year before NIV initiation. To our knowledge, this is the first study to describe risk factors for severe exacerbations in patients with overlap syndrome and to examine home NIV prescriptions in this specific population.

Previous RCTs have shown that NIV therapy in patients with hypercapnia and COPD prevented future exacerbations^32^ and reduced mortality.^33^ Retrospective studies in the United States have also shown an association of home NIV with lower readmission rates,^34^ and reduced risk of mortality, hospitalizations, and emergency department visits^35^ after hospital discharge. However, these studies have either excluded patients with comorbid OSA or did not include sleep apnea assessment. The results from the current study are consistent with available literature relating to COPD exacerbations and may be informative for overlap syndrome management. Unsurprisingly, severe exacerbations in the year before NIV were the most significant factor in predicting future exacerbations.^36^, ^37^, ^38^, ^39^, ^40^ This reinforces the importance of care management to prevent severe exacerbations and hospitalizations in the first instance because worsened prognosis and increased health care utilization tend to follow.^41^ Factors associated with increased exacerbation risk in patients with COPD alone were also identified in this analysis of COPD-OSA, including anxiety, asthma, heart failure, pneumonia, and mental disorders.^42^, ^43^, ^44^

The economic burden associated with overlap syndrome has been well described,^45^ but there is a lack of clinical outcome and health care utilization data for overlap syndrome treatment and adherence to home NIV therapy. Sterling et al^11^ reported a reduction in exacerbations, including hospitalization and emergency department visits, and health care costs in patients with overlap syndrome who were adherent to PAP. However, the study was restricted to CPAP and did not include bilevel PAP or NIV. Nearly one-quarter of this cohort had a CPAP claim in the year before NIV, but it may also be the case that they had an in-laboratory titration with failed CPAP and thus received NIV targeting OSA treatment. It is also likely that some patients were prescribed NIV due to chronic hypercapnia. Regardless, this indicates the importance of screening and treating OSA as part of COPD management due to the potential association of home NIV with exacerbation reduction. NIV for treatment of overlap syndrome may be targeted toward OSA in current clinical practice, but our results suggest it may also be considered to improve COPD symptoms and reduce exacerbation occurrence in this population. NIV eradicates apneic events and oxygen desaturations, allowing for better sleep quality and restoration,^46^ but also may help alleviate dyspnea, hypoventilation, hyperinflation, and hypercapnia, which have been shown to be associated with increased exacerbation risk.^47^

The observed increased risk associated with claims for maintenance and rescue medication is likely a proxy for disease severity and health care utilization, and not the result of medication usage. Snoring and obesity are often synonymous with a more severe and symptomatic OSA and chronic bronchitis phenotype; therefore, we hypothesized that the protective effect seen against exacerbations is a byproduct of a more pronounced clinical response and symptom improvements provided by NIV therapy. A high BMI has been linked to reduced exacerbation risk^48^ and, in general, individuals with emphysema-predominant COPD have a worse respiratory prognosis and may experience severe exacerbations more often than their higher BMI chronic bronchitis counterparts.^49^

The sample size of this real-world study is, to our knowledge, the largest published in this population and enhances the representation of a broad spectrum of patients with overlap syndrome receiving home NIV therapy in standard practice in the United States. The results from this study reflect the high prevalence of comorbid sleep apnea in patients with COPD who receive NIV therapy, highlighting the importance of screening for and treating sleep apnea as part of COPD disease management. This analysis included a 2-year observational period for all included patients, with the predictors selected from 1 year before NIV setup, and results reflective of the year after NIV initiation. Thus, the results indicate a rapid onset for the potential benefits of NIV treatment in patients with overlap syndrome with respect to COPD-related exacerbation occurrence. A novel aspect in the analytical approach of this study was the relative risk estimation from a retrospective cohort study using a binomial outcome. The reporting of RRs may be more informative and conservative from a public health perspective because epidemiologic and clinical research is largely grounded on the assessment of risk.^29^

The nature of a retrospective claims data analysis prevents the inclusion of laboratory values, medical test results, and modifiable or behavioral factors that may influence prognosis in COPD and contribute to exacerbation risk, including smoking status or smoking history, environmental exposures, physical activity, and alcohol or substance use. Therefore, it is possible that the exacerbation reduction was due to the improvement of chronic hypercapnic respiratory failure in addition to the treatment of OSA, among other factors. However, the lack of arterial blood gas levels limits our ability to quantify the relative contributions of each factor. These limitations in claims data also prevent researchers from drawing meaningful conclusions to inform public health interventions targeting behavior change at an individual, interpersonal, and policy level. This study lacked a comparator group of patients with overlap syndrome who were not on home NIV therapy, making it difficult to accurately interpret the impact of this therapy on the risk of severe exacerbations compared with patients who did not receive home NIV therapy. Furthermore, data on NIV usage and settings were not available. Understanding the prescribed and actual pressure settings in NIV would help fill some gaps from RCTs that failed to show improvement in COPD health outcomes during NIV therapy,^50^, ^51^, ^52^ because it has been hypothesized that the lack of benefit in these studies might have been due to inadequate pressure settings.^53^ Finally, the implementation of more robust statistical methods including causal analyses and predictive models are important areas for future research in this patient population. Subsequent studies should include comparator groups of patients who are on CPAP and patients without NIV therapy, and laboratory values like blood gases and apnea hypopnea index, to further discern the effect of home NIV on clinical outcomes in patients with overlap syndrome.

## Interpretation

This study provides insights into the characteristics of patients with COPD-OSA overlap syndrome using home NIV therapy and the factors associated with severe exacerbation occurrence in this population. The findings indicate the importance of preventing severe and moderate exacerbations from first occurring to prevent future severe exacerbations. Key comorbidities (eg, pneumonia, anxiety, heart failure, asthma, psychotic disorders) should be addressed in overlap syndrome management because they are associated with increased risk for severe exacerbations. Importantly, findings suggest that NIV prescription in patients with COPD and comorbid OSA was significantly associated with reduced exacerbation risk as soon as 1 year after NIV initiation. Finally, demographic factors associated with increased risk of severe exacerbations (eg, insurance type) stress the need for greater health equity efforts in the screening and treatment of COPD and sleep apnea.

To maximize the recognized benefits of home NIV therapy for patients with overlap syndrome, future research should focus on real-world clinical practice and incorporate a control group, adherence data, and analysis of therapy pressure settings. Parameters not captured in administrative claims such as lung function, physical activity, smoking history, COPD and OSA symptoms, and patient-reported outcomes (eg, quality of life) could also be included in future studies to assess their contribution to exacerbation risk. Ultimately, combining multiple data sources through a protected method like data tokenization could enable and provide the optimal database for a thorough assessment of exacerbation risk. The deeper understanding that results from this effort has the potential to help optimize access, timing, and therapeutic management of home NIV in patients with overlap syndrome.

## Funding/Support

This study was funded by 10.13039/100017647ResMed.

## Financial/Nonfinancial Disclosures

The authors have reported to *CHEST Pulmonary* the following: ResMed provided a philanthropic donation to UC San Diego, but A. M. has not received personal income from ResMed or medXcloud. A. M. is funded by the National Institutes of Health and has received income related to medical education from Zoll, Livanova, Powell Mansfield, and Eli Lilly. P. C. has an appointment to an endowed academic chair at the University of Sydney that was established from ResMed funding; has received research support from ResMed and SomnoMed; and is a consultant to ResMed, SomnoMed, and Sunrise Medical. J. L. P. is supported by the French National Research Agency in the framework of the Investissements d’Avenir program [Grant ANR-15-IDEX-02] and the e-Health and Integrated Care and Trajectories Medicine and MIAI Artificial Intelligence chairs of excellence from the Grenoble Alpes University [Grant ANR-19-P3IA-0003]; and has received lecture fees or conference traveling grants from ResMed, Philips, Jazz Pharmaceuticals, Agiradom, and Bioprojet. D. T., A. C., F. S.-K., and A. B. are all employees of ResMed. V. M. P is funded by NIH R61AG080606.

## Supplementary Data

e-Online Data

Internal Use
