# Supplementary material for: Treatment With Bilevel PAP Is Associated With a Reduction in Severe Exacerbations in COPD-OSA Overlap: A Retrospective Claims Analysis
Source: CHEST Pulm. 2024 Oct 28;3(1):100114. doi: 10.1016/j.chpulm.2024.100114 (PMC13418682; doi:10.1016/j.chpulm.2024.100114)
Supplement: e-Online Data [file mmc1.docx]

e-Table1: Diagnosis codes used to define COPD complexity level (Mapel et al. 2011)

| **Complexity Level** | **ICD-10 Diagnosis codes** |
| --- | --- |
| High complexity | Tuberculosis of lung (A15), aspergillosis (B44), Pneumocystosis (B59), malignant neoplasm of trachea (C33), malignant neoplasm of bronchus and lung (C34), malignant neoplasm of pleura (C384), secondary malignant neoplasm of pleura (C782), cystic fibrosis with pulmonary manifestations (E84), secondary polycythemia (D751), pulmonary embolism (I26), pulmonary heart diseases (I27). |
| Moderate complexity | Pneumonia due to SARS-associated coronavirus (J1281), viral pneumonia (J12), pneumonia due to streptococcus pneumoniae (J13), pneumonia due to Hemophilus influenzae (J14), bacterial pneumonia (J15), pneumonia due to other infectious organisms, not elsewhere classified (J16), cytomegaloviral pneumonitis (B250), whooping cough (A3791), pulmonary anthrax (A221), invasive pulmonary aspergillosis (B440), pneumonia in diseases classified elsewhere (J17), pneumonia, unspecified organism (J18), influenza due to unidentified influenza virus with pneumonia (J110), chronic obstructive pulmonary disease with (acute) exacerbation (J441), chronic obstructive pulmonary disease with (acute) lower respiratory infection (J440), emphysema, unspecified (J439), mild intermittent asthma with status asthmaticus (J4522), mild intermittent asthma with (acute) exacerbation (J4521), unspecified asthma with status asthmaticus (J45902), unspecified asthma with (acute) exacerbation (J45901), bronchiectasis, uncomplicated (J479), bronchiectasis with (acute) exacerbation (J471), farmer’s lung (J670), hypersensitivity pneumonitis due to unspecified organic dust (J679), pneumoconiosis due to asbestos and other mineral fibers (J61), Pneumonitis due to inhalation of food and vomit (J690), Pyothorax with fistula (J860), Pyothorax without fistula (J869), Pleural effusion, not elsewhere classified (J90), Spontaneous tension pneumothorax (J930), Other spontaneous pneumothorax (J931), Other pneumothorax and air leak (J938), Abscess of lung and mediastinum (J85), Hypostatic pneumonia, unspecified organism (J182), Chronic pulmonary edema (J811), Pulmonary fibrosis, unspecified (J8410), Other specified interstitial pulmonary diseases (J8489), Alveolar proteinosis (J8401), Idiopathic pulmonary hemosiderosis (J8403), Idiopathic interstitial pneumonia (J8411), Lymphoid interstitial pneumonia (J842), Other alveolar and parieto-alveolar conditions (J8409), Interstitial pulmonary disease, unspecified (J849), Pneumonia in diseases classified elsewhere (J17), Systemic sclerosis with lung involvement (M3481), Respiratory disorders in diseases classified elsewhere (J99), Pulmonary collapse (J981), Pulmonary eosinophilia, not elsewhere classified (J82), Acute pulmonary edema (J810), Acute postprocedural respiratory failure (J95821), Acute respiratory failure, unspecified whether with hypoxia or hypercapnia (J9600), Acute pulmonary insufficiency following thoracic surgery (J951), Acute pulmonary insufficiency following nonthoracic surgery (J952), Chronic pulmonary insufficiency following surgery (J953), Acute and chronic postprocedural respiratory failure (J95822), Acute and chronic respiratory failure, unspecified whether with hypoxia or hypercapnia (J9620), Allergic bronchopulmonary aspergillosis (B4481), Respiratory failure, unspecified, unspecified whether with hypoxia or hypercapnia (J9690), Acute respiratory distress syndrome (J80), Chronic respiratory failure, unspecified whether with hypoxia or hypercapnia (J9610), Acute and chronic respiratory failure, unspecified whether with hypoxia or hypercapnia (J9620), Unspecified tracheostomy complication (J9500), Infection of tracheostomy stoma (J9502), Malfunction of tracheostomy stoma (J9503), Other tracheostomy complication (J9509), Cyanosis (R230), Orthopnea (R0601), Hemoptysis (R042), Hemorrhage from respiratory passages, unspecified (R049), Acute idiopathic pulmonary hemorrhage in infants (R0481), Hemorrhage from other sites in respiratory passages (R0489), Respiratory arrest (R092), Tracheostomy status (Z930), Acquired absence of lung [part of] (Z902), Dependence on respirator (Z991), Dependence on supplemental oxygen (Z9981), Encounter for attention to tracheostomy (Z430). |

A patient has high complexity COPD if he or she has been identified as having COPD and has any of the following diagnoses or services during the year prior to home NIV initiation that are designated as high. A patient has moderate complexity COPD if he or she has been identified as having COPD, has not been designated as having high complexity COPD, and has had any of the following diagnoses or services during the year prior to home NIV initiation that are designated as moderate. If a COPD patient did not have any comorbid condition for high or moderate complexity, they were classified as low complexity.

e-Table 2. Severe exacerbation occurrence distribution before and after NIV initiation.

|  |  | **Severe exacerbations 1Y After NIV** | |  |
| --- | --- | --- | --- | --- |
|  |  | **0** | **≥1** | **Total** |
| **Severe exacerbations 1Y Prior to NIV** | **0** | 20,835 | 704 | 21,539 |
|  | **≥1** | 1,742 | 711 | 2,453 |
| **Total** | 22,577 | 1,415 | 23,992 |  |

**FIGURE LEGENDS**

**e-Figure 1.** Distribution of severe exacerbations. Baseline = 1 year prior to NIV index. Follow-up = 1 year after NIV index.
